# Chemical composition, biological activities, and quality standards of hawthorn leaves used in traditional Chinese medicine: a comprehensive review

**DOI:** 10.3389/fphar.2023.1275244

**Published:** 2023-10-20

**Authors:** Wenjing Guo, Tingting Shao, Yu Peng, Haitao Wang, Zhe-Sheng Chen, Haixiang Su

**Affiliations:** ^1^ Gansu University of Chinese Medicine, Lanzhou, China; ^2^ Gansu Provincial Cancer Hospital, Gansu Provincial Academic Institute for Medical Research, Lanzhou, China; ^3^ The First Hospital of Lanzhou University, Lanzhou, China; ^4^ Department of Pharmaceutical Sciences, College of Pharmacy and Health Sciences, St. John’s University, Queens, NY, United States

**Keywords:** hawthorn leaves, chemical composition, quality standard, biological activity, traditional Chinese medicine

## Abstract

Hawthorn leaves also known as crataegi foilum, are a combination of botanical drugs used commonly in Traditional Chinese Medicine. Hawthorn, the plant from which hawthorn leaves are prepared, is distributed in Northeast China, North China, and other regions in China. Hawthorn leaves are known to activate blood circulation and eliminate stasis, invigorating Qi, eliminating turbidity, and reducing the levels of lipids. So far, over a hundred compounds have been isolated from hawthorn leaves, including flavonoids, terpenoids, lignans, organic acids, and nitrogenous compounds. Hawthorn leaves are used for the treatment of hypertension, protecting against ischemic injury, angina, hyperglycemia, hyperlipidemia, and certain other conditions. Several of the currently available clinical preparations also use hawthorn leaves as raw materials, such as Yixintong capsules, Xinan capsules, etc. The present report systematically reviews the chemical composition, biological activities, and quality standards of hawthorn leaves, to provide a scientific basis and reference for detailed research on hawthorn leaves.

## 1 Introduction

Hawthorn leaves are a combination of the dried leaves of the Rosaceae plants, i.e., *Crataegus pinnatifida* Bge. or *Crataegus pinnatifida* Bge. var. m*ajor* N. E. Br., used commonly in Traditional Chinese Medicine. Hawthorn is cultivated mainly in Northeast China, Beijing, Tianjin, Liaoning, Hebei, Taihang Mountain Area, and Shandong in China. They are harvested during the summer and autumn seasons. Hawthorn leaves appear broadly ovoid in shape, measuring 6–12 cm in length and 5–8 cm in width, apex tapering, broadly wedge-shaped at the base with 2–6 pinnae lobes, and sharply heavy serrations at edges. They have a green to brownish-yellow color, the petioles measure 2–6 cm in length, and the stipules are ovoid to ovoid-lanceolate in shape. According to the Chinese Pharmacopoeia (2020 Edition), hawthorn leaves activate blood circulation and eliminates stasis, thereby invigorating Qi to pass through the blood and also eliminating turbidity, resulting in lowered lipid levels.

The compounds that have been isolated from hawthorn leaves to date include flavonoids, pentacyclic triterpenes, monoterpenes, sesquiterpenes, lignans, and organic acids (Ying, 2007; Hao, 2008; Gao, 2009; Wang, 2020). Modern pharmacological studies have demonstrated that hawthorn leaves exhibit various pharmacological activities, such as hypolipidemic (Yang et al., 2007; Liu and Yang, 2008; Diao et al., 2020), antithrombotic (Li et al., 1988), hypotensive (Huang, 2001; Chang et al., 2002), anti-myocardial ischemia (Ji et al., 2005; Cui et al., 2006; Kan et al., 2007; Zhong et al., 2014; Xue et al., 2020), anti-arrhythmic (Wu et al., 2002), anti-inflammatory (Quan and Guan, 2006; Wang et al., 2012), and glucose-lowering (Ye et al., 2005; Zhang and Zhang, 2015; Zhou et al., 2016; Chen et al., 2017) activities. Recently, hawthorn leaves have attracted great attention for their usefulness in the clinical treatment of cardiovascular diseases, and several formulations containing hawthorn leaves as component, such as Yixintong capsules (the main component of which is the total flavonoids from hawthorn leaves) (Qian, 2001) and Xinan capsules (the main component of which is the flavonoid extracted from hawthorn leaves), have been used clinically to treat various cardiovascular diseases, such as hyperlipidemia, coronary heart disease, angina pectoris, and arrhythmias. Despite the usefulness and popularity of hawthorn leaves, the existing literature does not contain any comprehensive review of the research on hawthorn leaves from the three aspects of chemical composition, pharmacological activity, and quality standard.

Therefore, the present review article was prepared to systematically summarize the research progress of research on hawthorn leaves from the aspects of chemical composition and biological activity to obtain a further comprehensive understanding of hawthorn leaves and thereby strengthen the foundation for the clinical application of hawthorn leaves. The other objective was to comprehensively summarize the quality standard for hawthorn leaves to guide the research in the direction of improving the quality of hawthorn leaves. The present review is, therefore, expected to provide a scientific basis for detailed research on hawthorn leaves and the healthy development of the related industry.

## 2 Chemical composition

Numerous studies have been conducted to date on the chemical composition of hawthorn leaves. The following sections in the present article systematically summarize a few of these studies that have been conducted in domestic and international research.

### 2.1 Flavonoids

**FIGURE 1:**
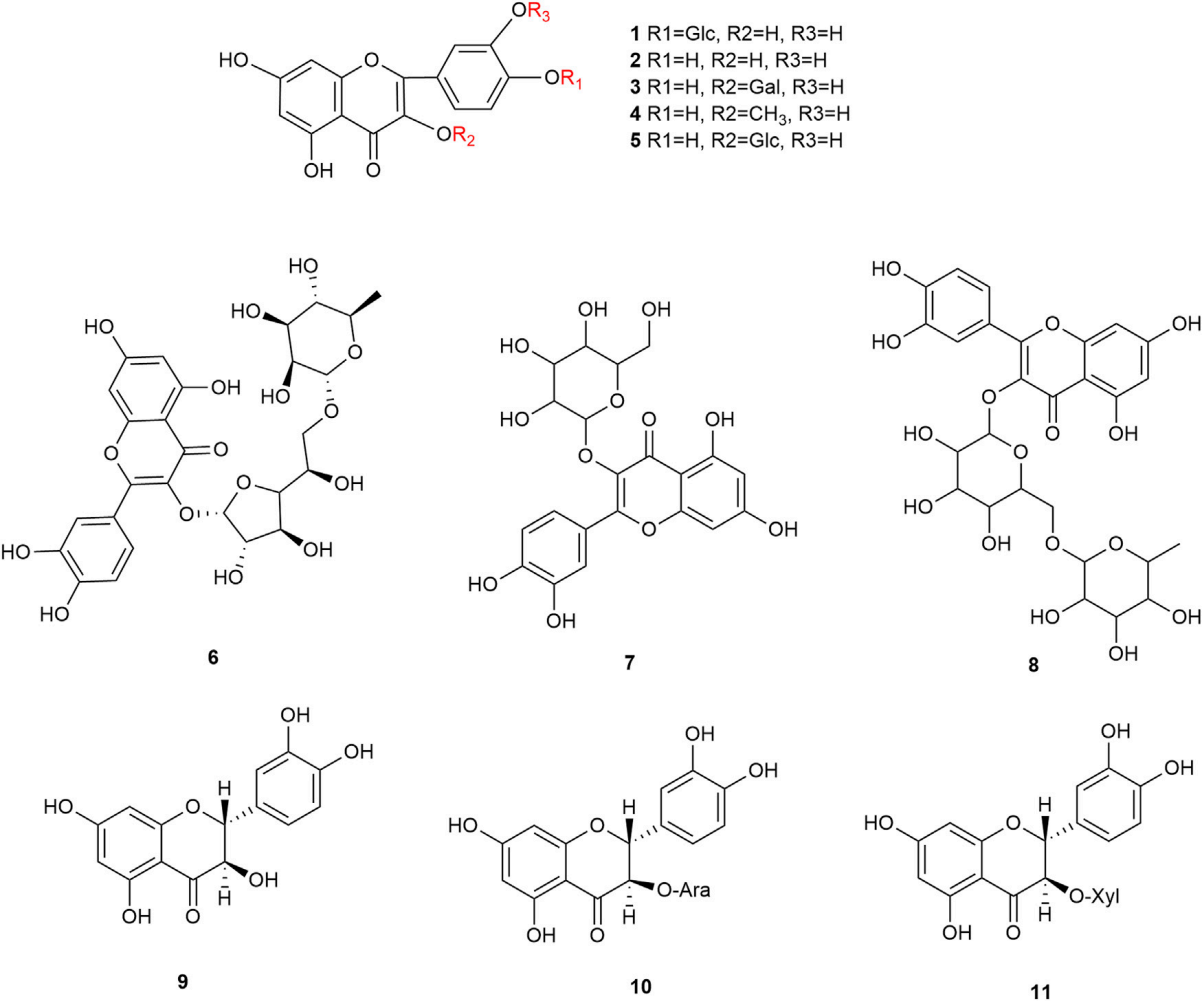
The chemical structures of quercetin flavonoids in hawthorn leaves.

**FIGURE 2:**
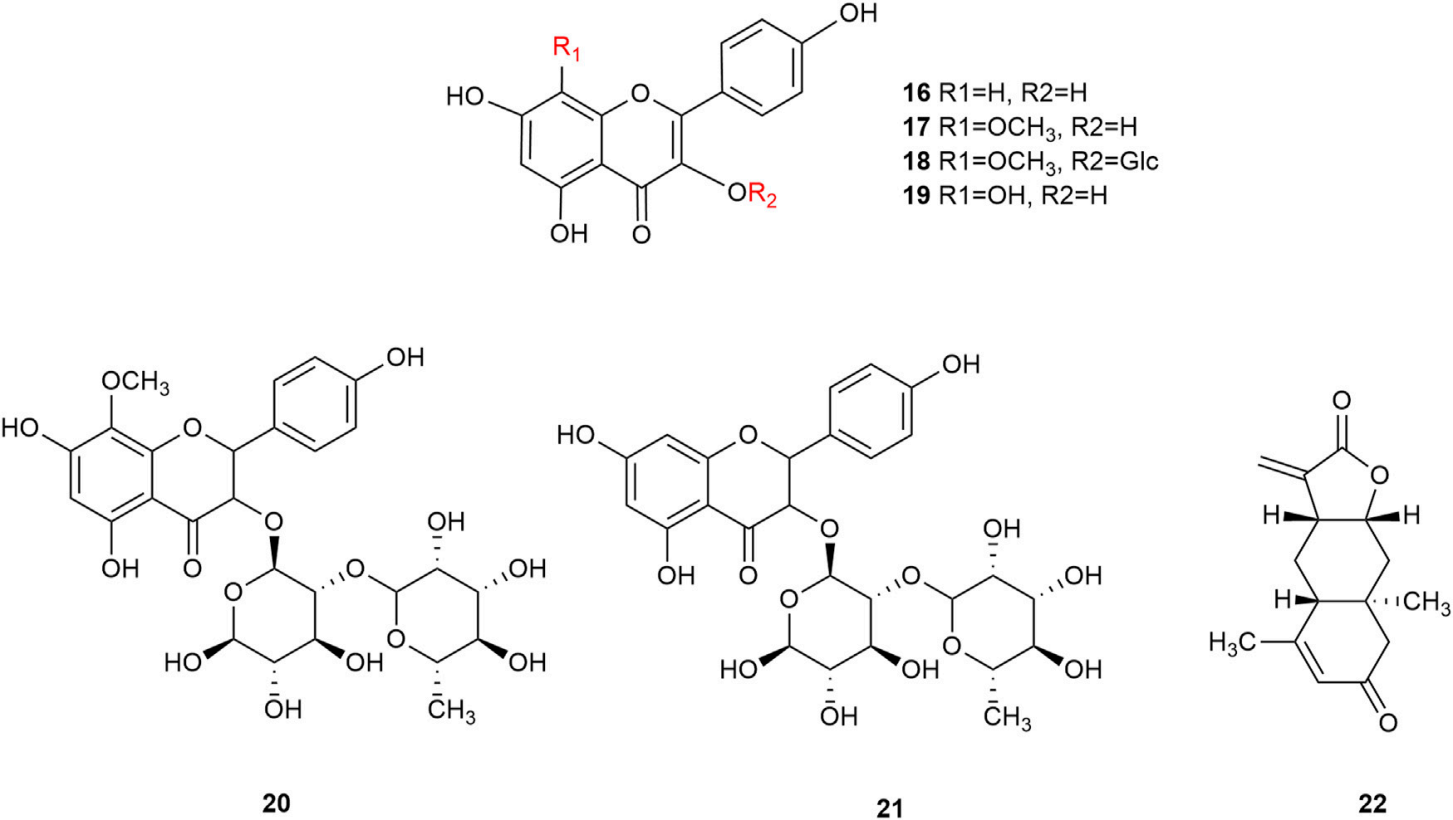
The chemical structures of kaempferol flavonoids in hawthorn leaves.

**FIGURE 3:**
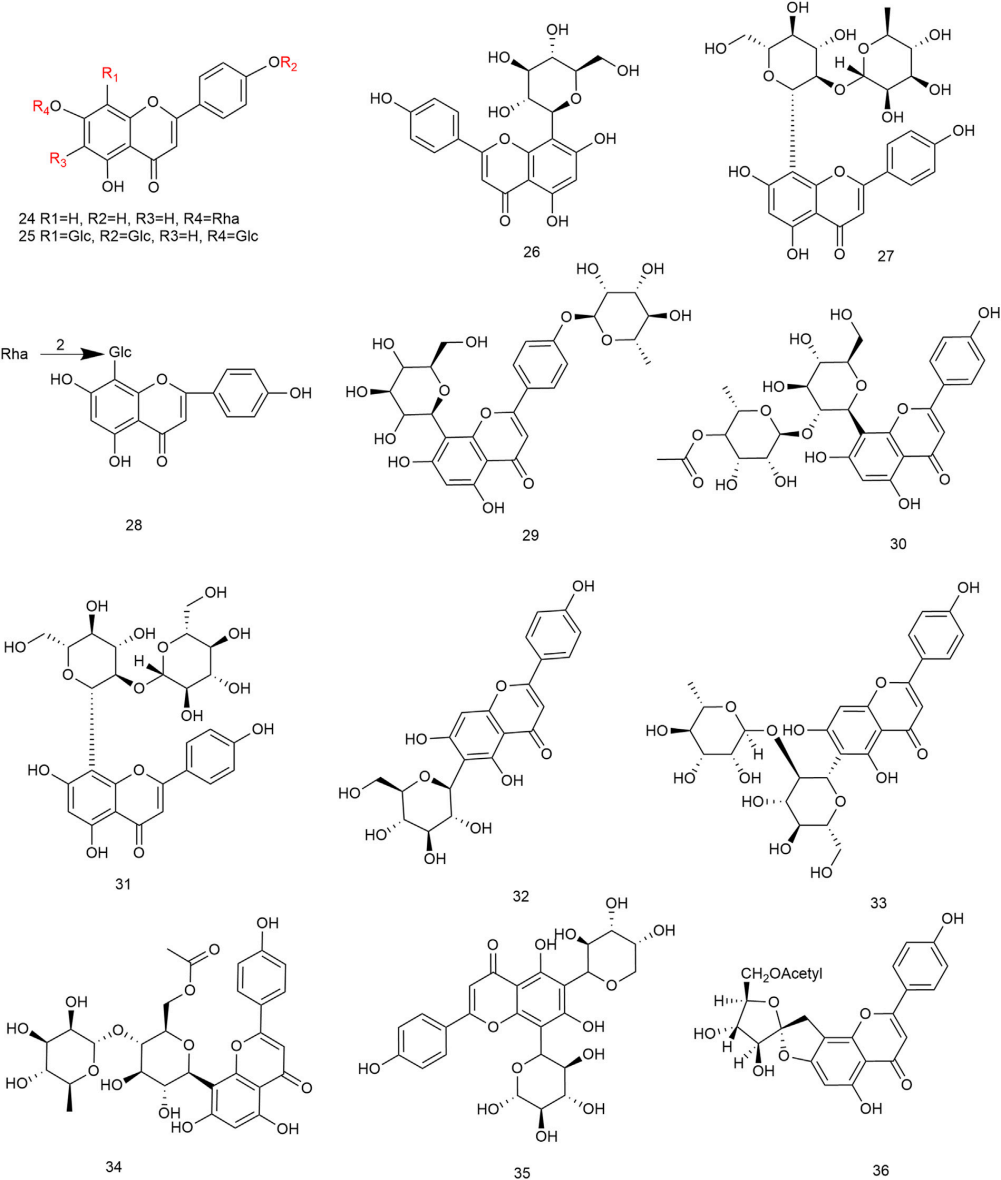
The chemical structures of apigenin flavonoids in hawthorn leaves.

**FIGURE 4:**
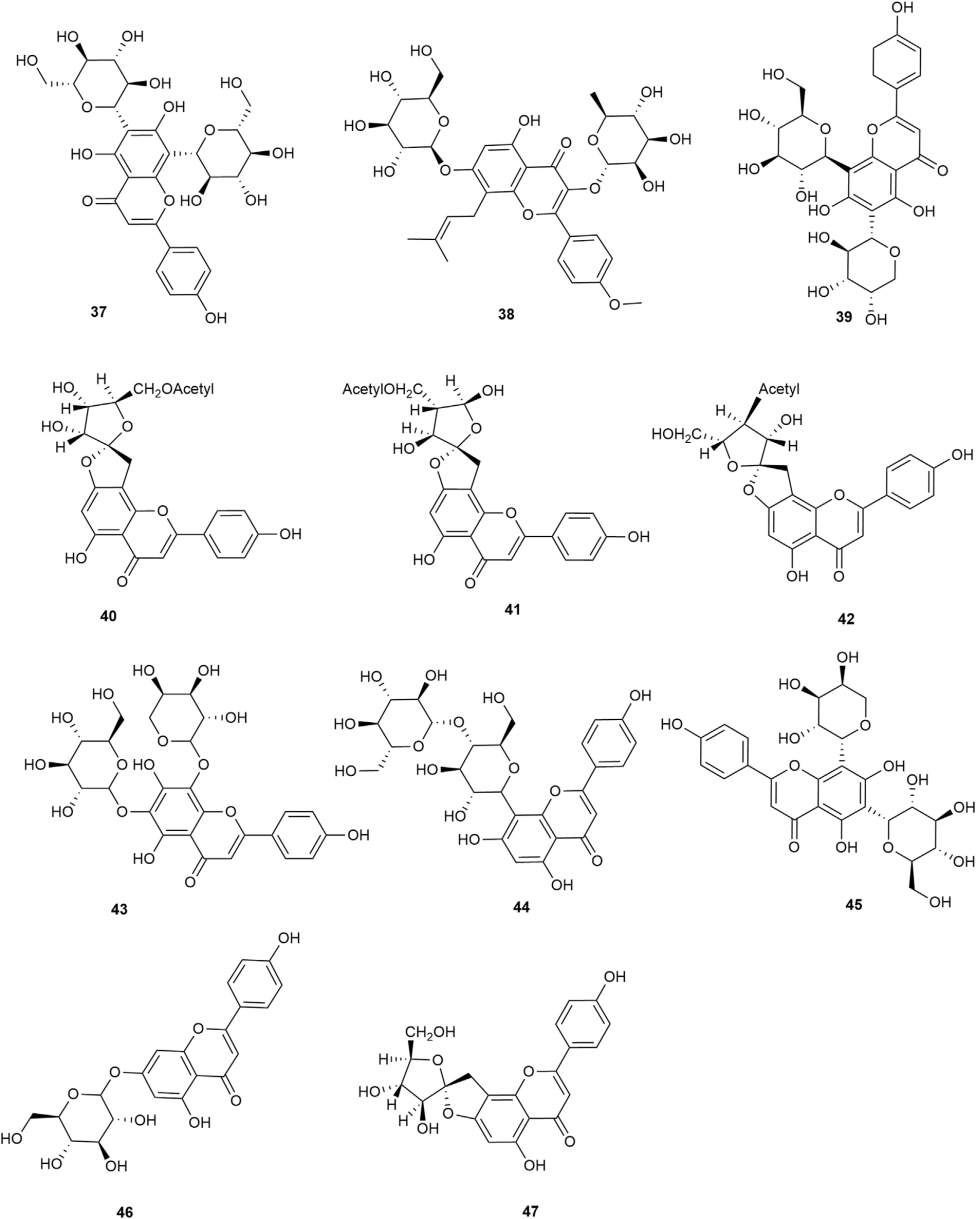
The chemical structures of apigenin flavonoids in hawthorn leaves.

**FIGURE 5:**
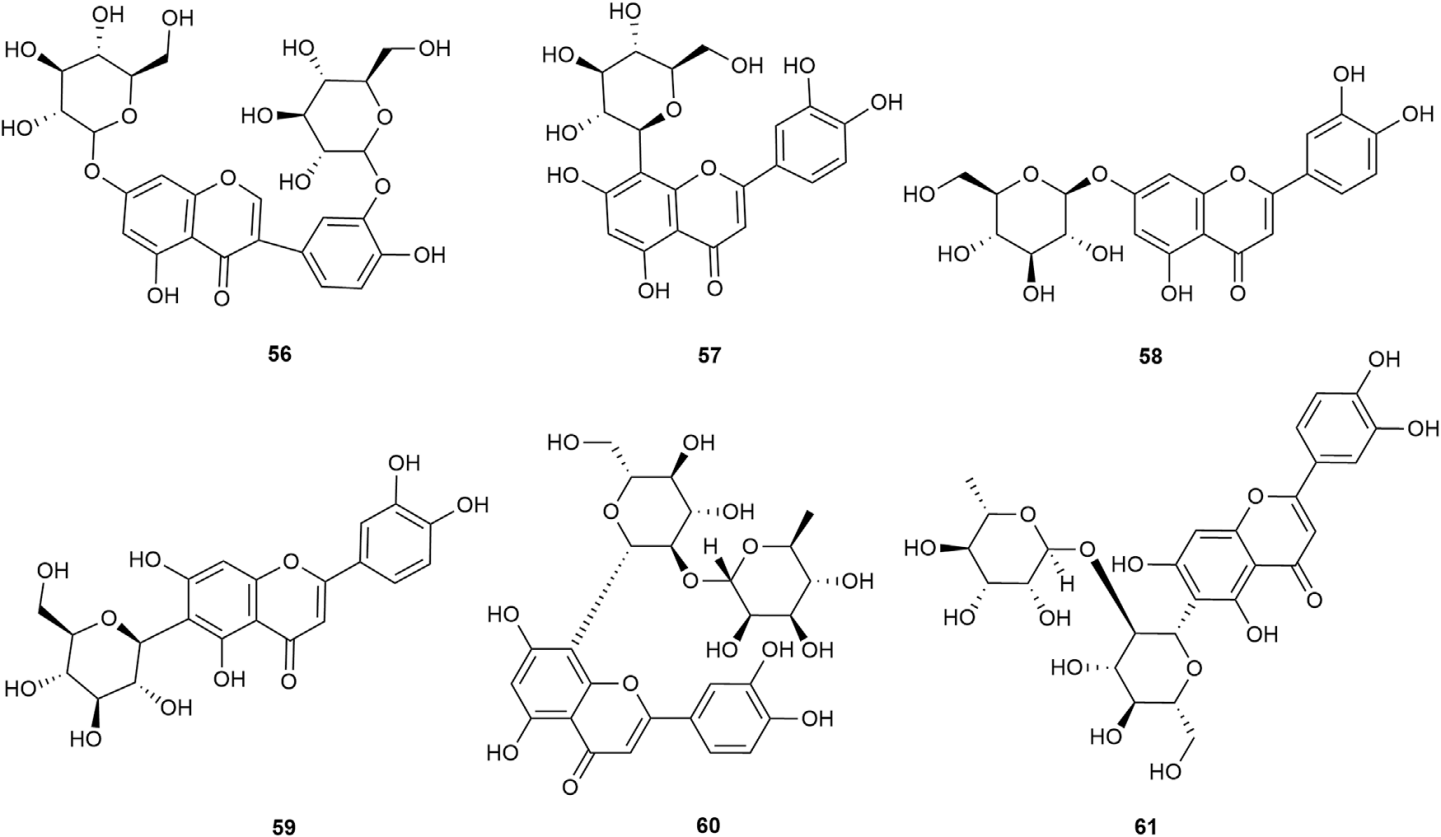
The chemical structures of luteolin flavonoids in hawthorn leaves.

**FIGURE 6:**
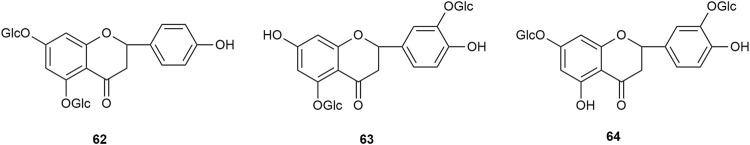
The chemical structures of dihydroflavonoid flavonoids in hawthorn leaves.

**FIGURE 7:**
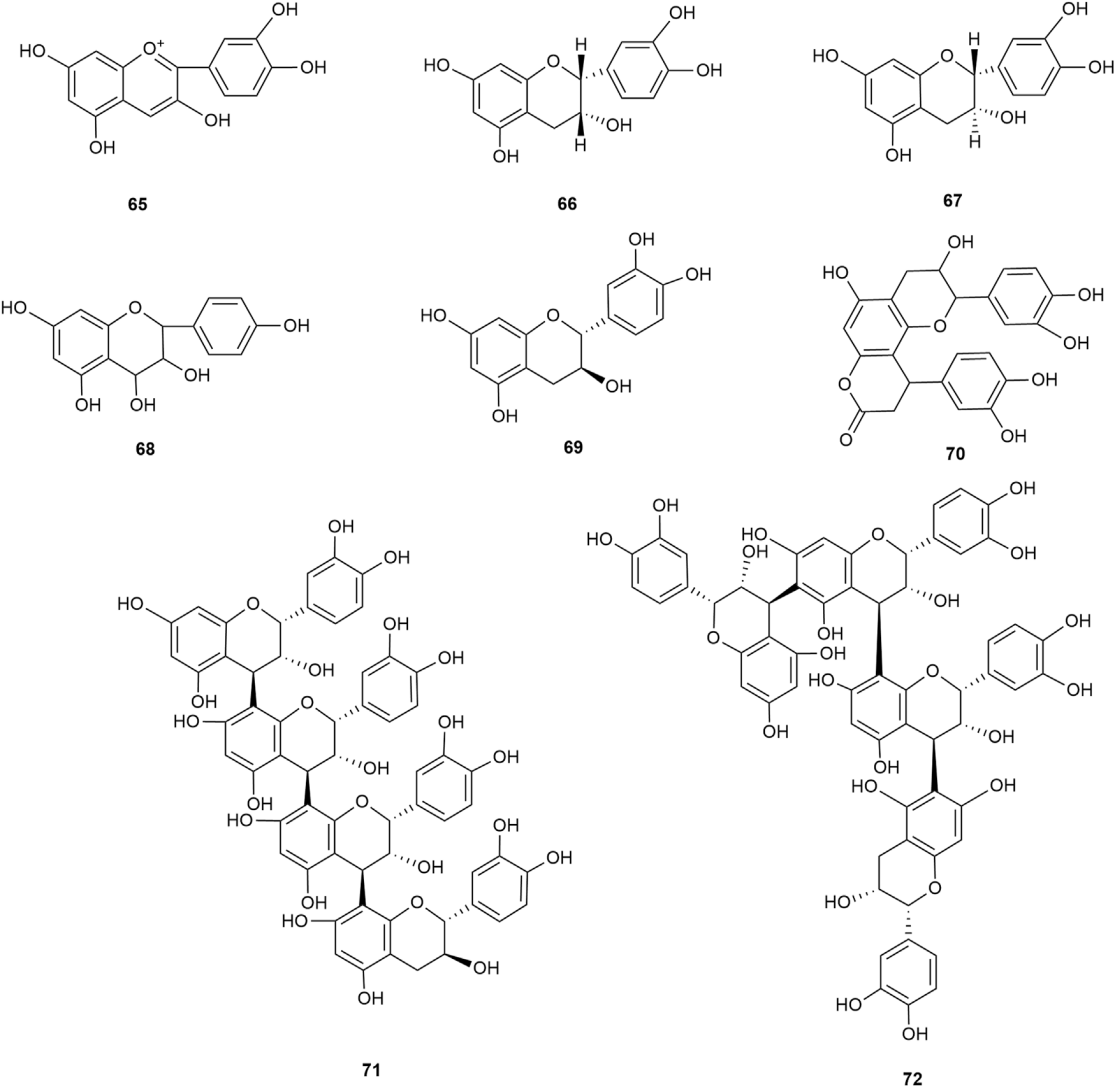
The chemical structures of flavans and their polymers in hawthorn leaves.

**FIGURE 8:**
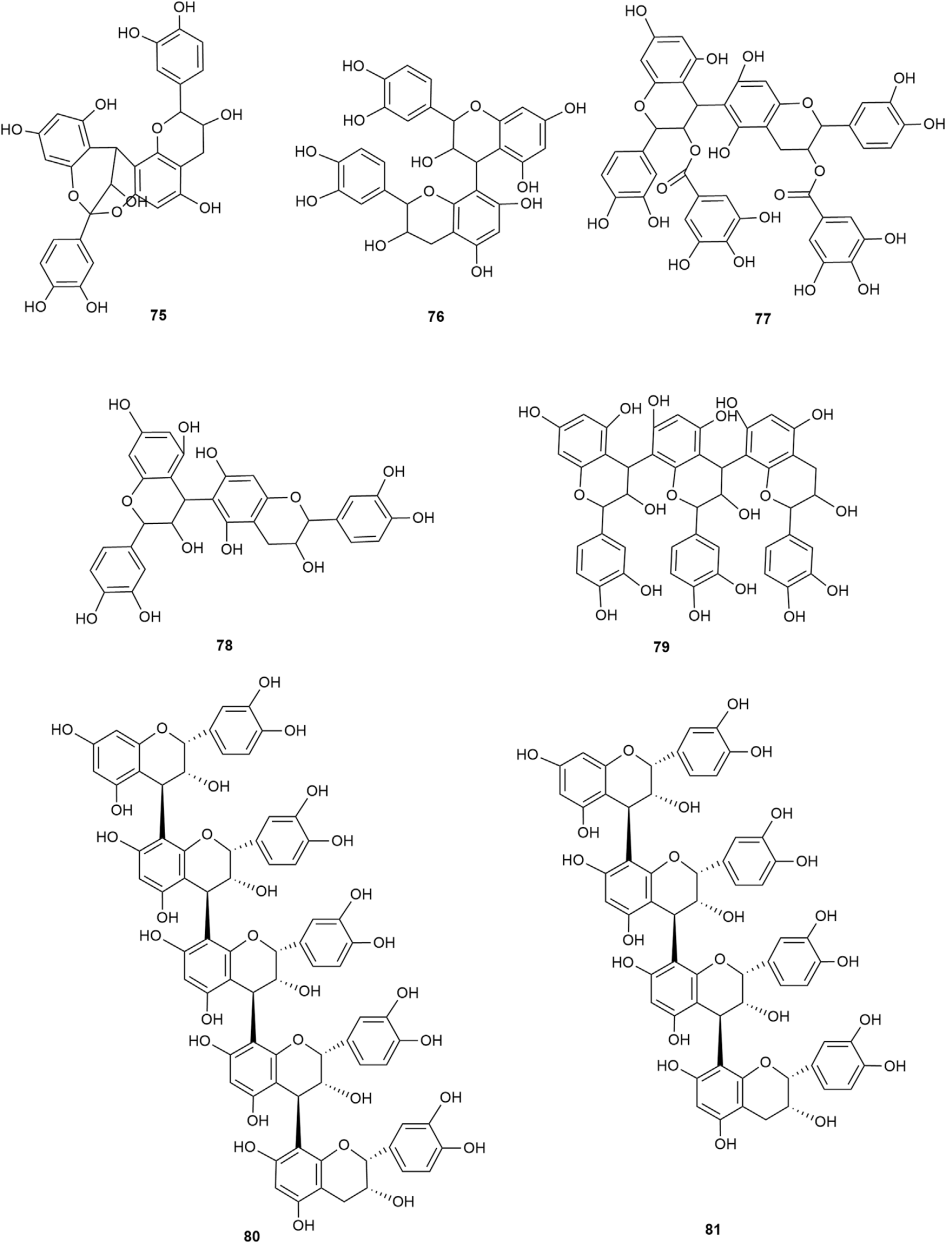
The chemical structures of anthocyanins in hawthorn leaves.

Flavonoids are the main components of hawthorn leaves. So far, over 60 flavonoids and flavonoid glycosides have been identified and isolated from hawthorn leaves. Among these, four flavonoids are specific to hawthorn leaves–vitexin-4″-O-rhamnoside, 8-C-B-D(2″-O-acetyl)-glucofunoside apigenin, eriodictyol-5,3′-diglucoside, and eriodictyol-7,3′-diglucoside. According to the parent structure, the flavonoids in hawthorn leaves are divided into flavones, flavonols, dihydroflavones, dihydroflavonols, flavanes, and anthocyanins. The flavonoid glycosides in hawthorn leaves are classified into five main classes based on their constituting aglycone structure–quercetin flavonoids, apigenin flavonoids, kaempferol flavonoids, luteolin flavonoids, and dihydroflavonoid flavonoids. The monomer species of flavonoids are presented in Supplementary Table S1–S7, (Sun et al., 2014; Ding et al., 1990; Chen, 2006; Chen et al., 2006; Wang, 2020; Du et al., 2022; Si et al., 1998; Shahat et al., 1998; Zhang and Xu, 2001a; Zhang and Xu, 1999a; Zhao et al., 2002; Zhang and Xu, 1999b; Xu et al.,1985; Liu et al., 2007; Hao, 2008; Nikolov and Litvinenko, 1973; Zhang and Xu, 2001b; Svedstrom et al., 2002) and the structural formulas of these monomers are presented in Figure 1 Figure 2, Figure 3, Figure 4, Figure 5, Figure 6, Figure 7 to Figure 8.

### 2.2 Terpenoids

**FIGURE 9:**
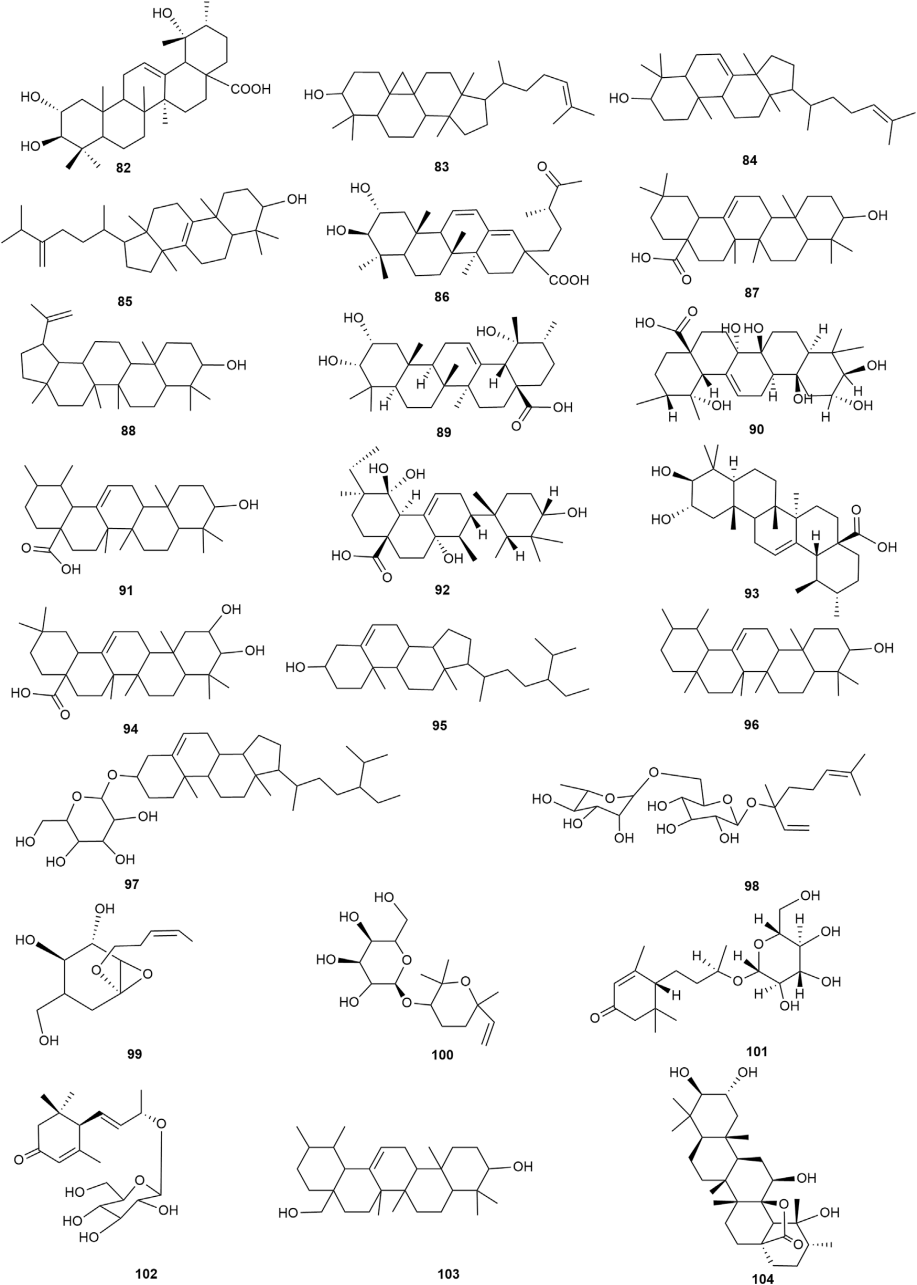
The chemical structures of triterpenoids and steroids in hawthorn leaves.

**FIGURE 10:**
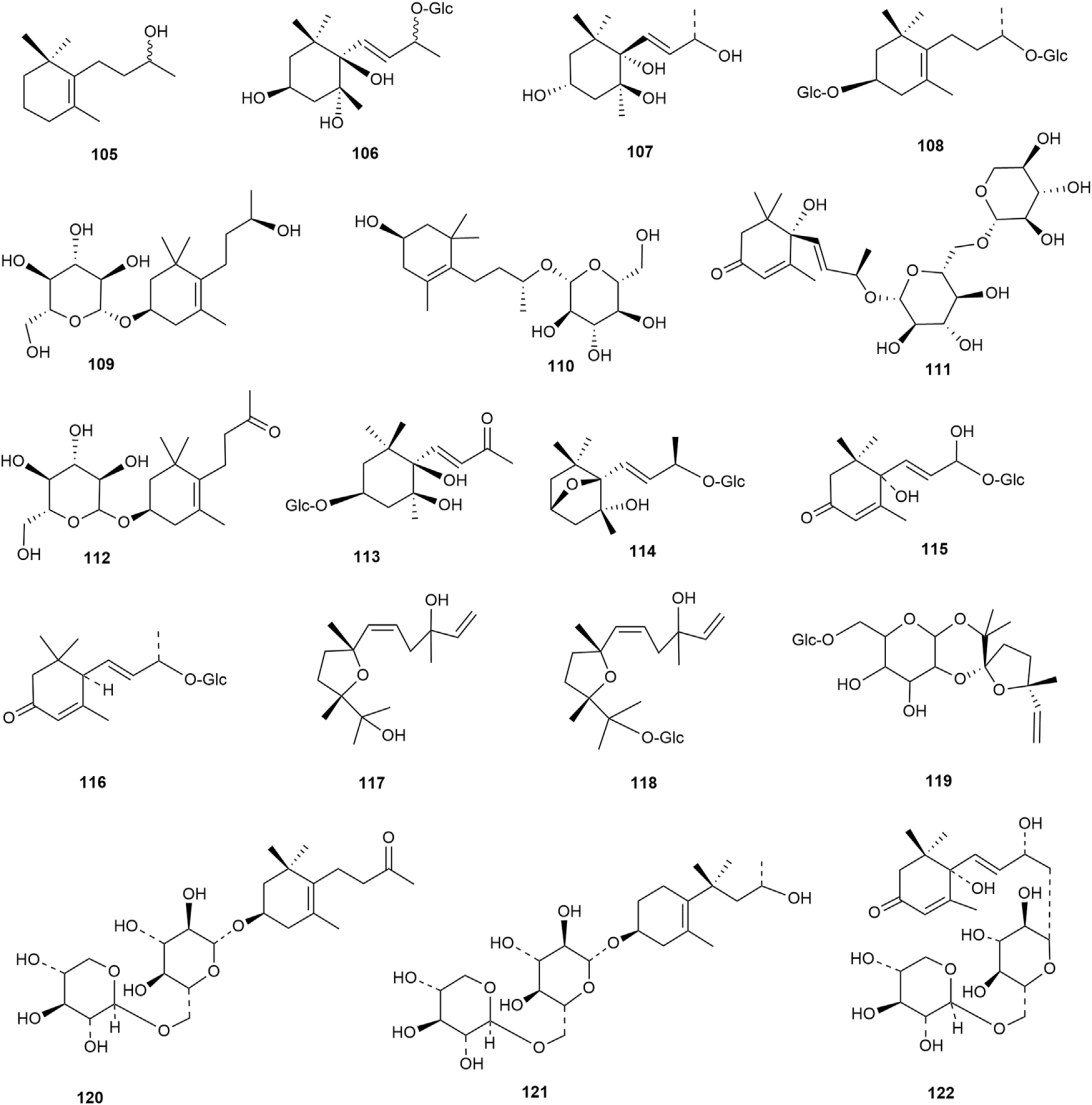
The chemical structures of monoterpenes and sesquiterpenes in hawthorn leaves.

Terpenoids are also among the main components of hawthorn leaves, and over 30 terpenoids have been identified and isolated from hawthorn leaves to date. According to the chemical structure, terpenoids are mainly divided into steroids, triterpenes, monoterpenes, and sesquiterpenes, among which monoterpenes and sesquiterpenoids are the most abundant. Among these, 2α,3β,19α-trihydroxyl ursolic acid, which is a pentacyclic triterpenoid, is present specifically in hawthorn leaves and is also the only triterpenoid isolated from hawthorn leaves to date. The common steroidal compounds present in hawthorn leaves include cycloartenol, β-sitosterol, and butyrospermol, among others. The monomer species of terpenoids are presented in Supplementary Table S8, S9, (Song et al., 2006; Garcia et al.,1997; Huang et al., 2010; Zhang and Xu, 1999a; Gao, 2009; Wang, 2020; Duan et al., 2021; Si et al., 1998; Chen et al., 2008; Hao, 2008; Shi and Ding, 2000) and the structural formulas of these monomers are presented in Figure 9 and Figure 10.

### 2.3 Lignan

**FIGURE 11:**
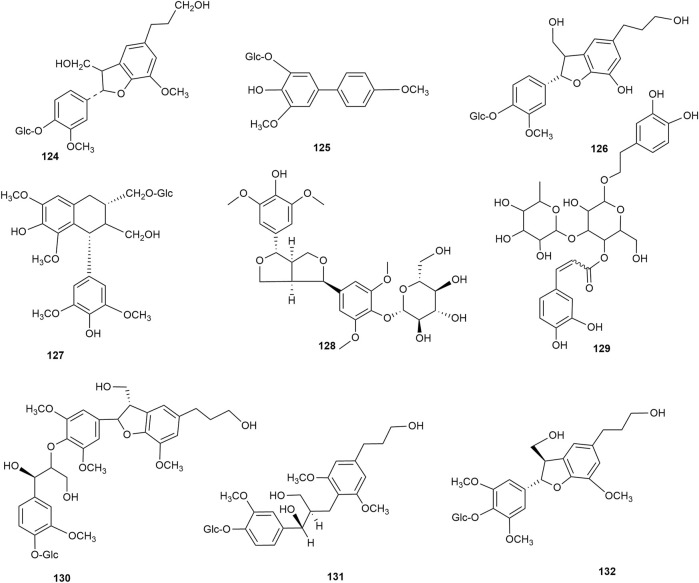
The chemical structures of lignan compounds in hawthorn leaves.

Lignan is a natural compound formed mainly of the dimers of phenylpropanoid units (C6-C3 monomers), with a few trimers and tetramers. According to the existing literature, hawthorn leaves contain 9 lignan compounds. Dongfang Hao identified the lignan 2,3-dihydro-2-(4′-O-β-D-glucopyranosyl-3′-methoxy-phenyl)-3-hydroxymethyl-5-(3-hydroxpropyl)-7-methoxybenzofuran in hawthorn leaves using various chromatographic methods, such as silica gel column chromatography, Spehdaxe LH-20 column chromatography, macroporous resin chromatography, and semi-preparative reverse HPLC (Hao, 2008). Jia Chen et al. isolated shanyenoside A from hawthorn leaves using silica gel column chromatography, polyamide column chromatography, and Sephadex LH 20, followed by its identification using physicochemical and spectral methods (Chen et al., 2006). Pin Yi Gao et al. isolated the lignan compounds tortoside A, (7S,8R)-urolignoside, (−)-2a-O-(β-D-glucopyranosyl)-lyoniresinol, verbascoside, acernikol-4″-O-β-D-glucopyranoside, erythron-1-(4-O-β-D-glucopyranosyl-3-methoxyphenyl)-2-[4-(3-hydroxypropyl)-2,6-dimethoxyphenoxy]-1,3-propanediol, and (7S,8R)-5-methoxydihydrodehydrodiconiferyl alcohol-4-O-β-D-glucopyranoside from hawthorn leaves (Gao et al., 2010). The monomer species of ligan compounds are listed in Supplementary Table S10, and the structural formulas of these monomers are presented in Figure 11.

### 2.4 Organic acid

**FIGURE 12:**
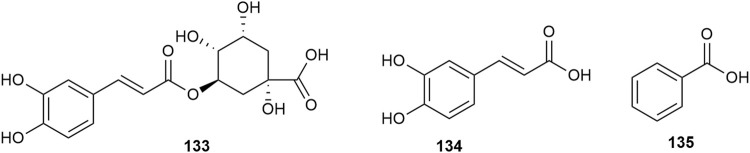
The chemical structures of organic acid compounds in hawthorn leaves.

Hawthorn leaves contain various organic acid compounds, among which benzoid acid was the first one to be isolated and reported by Xiaoxiao Huang et al., in 2010 (Huang et al., 2010). The organic compound chlorogenic acid in hawthorn leaves was isolated by Ting Gao (Gao, 2009), and caffeic acid was isolated by Lingdi Wang et al. (Wang et al., 2011). The monomer species of organic acid compounds are listed in Supplementary Table S11, and the structural formulas of these monomers are presented in Figure 12. Shufang Liang et al. reported that hawthorn leaves had 17 types of amino acids, with aspartic acid and glutamic acid accounting for the highest proportions and methionine and histidine accounting for the lowest content, while alanine, isoleucine, and leucine are also present in high amounts (Liang et al., 1996). The different types of amino acids in hawthorn leaves listed in the order of decreasing proportion are as follows: glutamic acid > aspartic acid > isoleucine > leucine > alanine > serine > glycine > threonine > valine > cystine, lysine, proline > arginine, phenylalanine > tyrosine > histidine > methionine. When the contents of these 17 amino acids in hawthorn leaves were measured, it was revealed that the contents of aspartic acid, glutamic acid, cystine, alanine, and isoleucine in hawthorn leaves were much higher than those in hawthorn fruit. These amino acid species are listed in Supplementary Table S12.

### 2.5 Nitrogenous compounds

**FIGURE 13:**
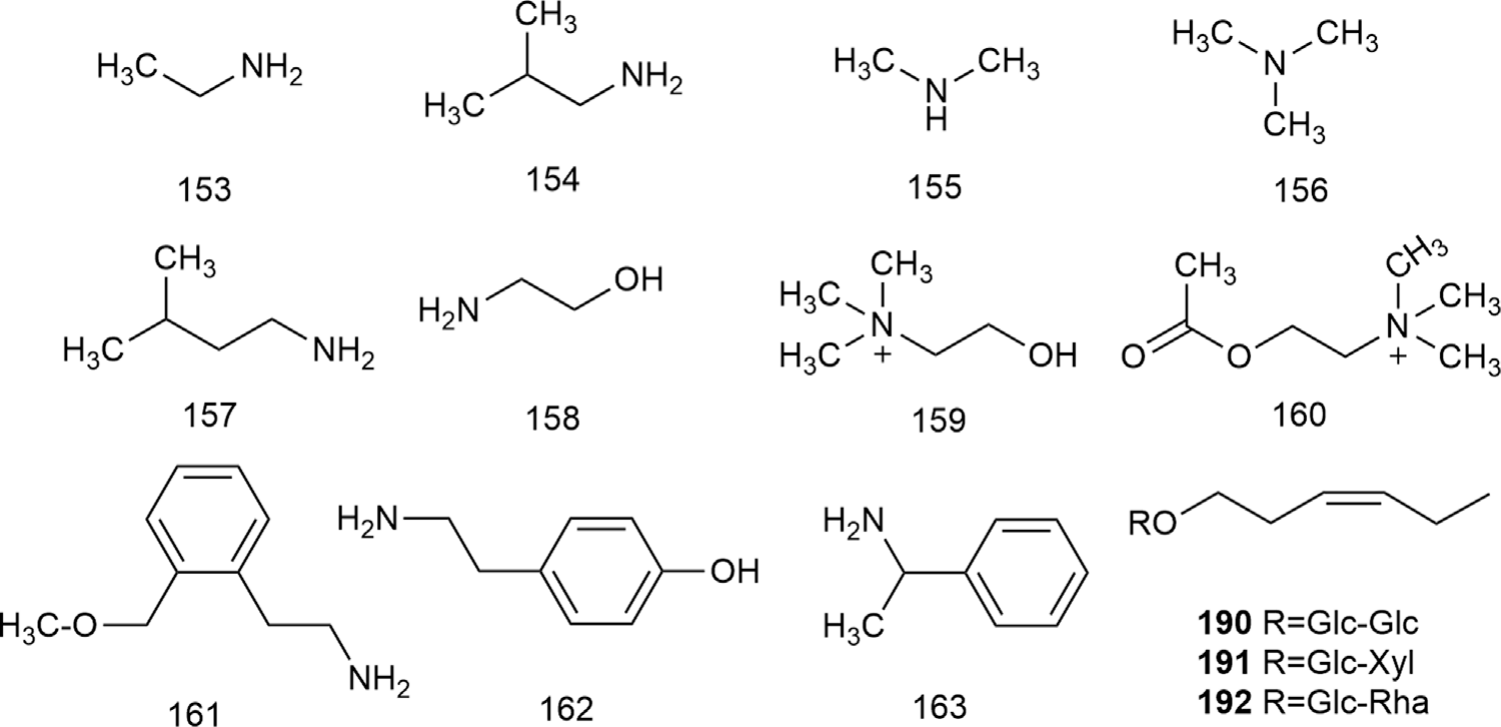
The chemical structures of nitrogen containing compounds and other classes of compounds in hawthorn leaves.

Nitrogenous compounds are organic compounds containing carbon–nitrogen bonds in their molecular structures and include amines, nitrogen heterocycles, nitriles, and nitro compounds. So far, over ten nitrogenous compounds have been identified and isolated from hawthorn leaves. Hobbs et al. reported the presence of nitrogen-containing compounds such as dimethylamine, trimethylamine, isoamylamine, ethanolamine, choline, acetylcholine, and spermindine in hawthorn leaves. In addition, the authors reported the presence of organic amines such as O-methoxyphenethylamine, tyramine, isobutylamine, ethylamine, and phenylethylamine in hawthorn leaves. The monomer species of nitrogenous compounds are listed in Supplementary Table S13, (Foster and Hobbs, 1994) and the structural formulas of these monomers are presented in Figure 13.

### 2.6 Trace elements

Trace elements usually refer to elements that account for less than 1/10000^th^ of the total weight of the human body and are required in an amount of less than 100 mg per person per day. Although the demand for trace elements is small, these elements are highly important as nutrients for maintaining the normal physiological functions of human life. Trace elements are available in a wide variety, and based on their functional role, these are divided into essential trace elements and non-essential trace elements. Hawthorn leaves are rich in trace elements. Zhongjie Zhao et al. determined 25 trace elements in the composition hawthorn leaves using inductively coupled plasma atomic emission spectrometry (ICP-AES). The authors, based on the results obtained by averaging two measured data values, identified 10 essential trace elements in hawthorn leaves, namely, Cu, Zn, Fe, Mn, Sr, V, Co, Ni, Cr, and Mo. Among the 25 trace elements reported to date in hawthorn leaves, Fe, Ca, Mg, K, *P*, and Al account for the highest contents, while Co, Li, Be, Cd, As, and Hg, which are non-toxic to the human body, are present in low contents. Zhongjie Zhao et al. also determined the contents of the trace elements they identified in hawthorn fruit and reported that most trace elements in hawthorn leaves have contents higher, in general, than their contents in hawthorn fruit (Zhao et al., 1992). These trace elements are listed in Supplementary Table S14.

### 2.7 Other compounds

In addition to the compounds stated above, hawthorn leaves also contain certain other compounds, for example, (Z)-3-hexenyl-O-β-D-glucopyranosyl-(1''→6′)-β-D-glucopyranoside, (Z)-3-hexenyl-O-β-D-xylopyranosyl-(1''→6′)-β-D-glucopyranoside, and (Z)-3-hexenyl-O-β-D-rhamnopyranosyl-(1''→6′)-β-D-glucopyranoside, which were identified and reported by S. J. Song et al. (Song et al., 2011). All such compounds reported to date are listed in Supplementary Table S15, and the structural formulas of these compounds are presented in Figure 13.

In addition, hawthorn leaves contain nutrients such as proteins and vitamins, including vitamin C, vitamin B_1_, vitamin B_2_, and niacin (Liang et al., 1996). Jiali Sun et al. determined the content of vitamin C in hawthorn leaves using spectrophotometry and 2,4-dinitrophenylhydrazine colorimetry and reported that at maturity, the vitamin C content in hawthorn leaves was nearly 10 times higher than that in hawthorn fruit, the hawthorn leaves collected at different growth periods until just before defoliation. Moreover, in the dried hawthorn leaves stored at room temperature, vitamin C content reportedly decreases as the preservation duration increases and was also affected by the different treatment methods used for drying (Sun et al., 1991).

## 3 Biological activity

### 3.1 Blood pressure lowering

The total flavonoids, proanthocyanidin, triterpenoid acid extracts, and polysaccharide active components isolated from hawthorn leaves have exhibited anti-hypotensive effects. According to studies, when different animals were subjected to chronic consumption of flavonoids, oligomeric proanthocyanidins, triterpenoid acid extracts, and polysaccharide active ingredients, their blood pressure reduced in a dose-dependent manner, and the peripheral vasodilation was reported as the main mechanism underlying the antihypertensive effect of these compounds (Huang, 2001; Chang et al., 2002). Yixintong capsules, a formulation of Traditional Chinese Medicine, are prepared from the flavonoids extract of hawthorn leaves and are used clinically for lowering blood pressure and maintaining blood pressure stability. Weiping Qian observed that perfusing isolated frog hearts with 5 mg/mL, 10 mg/mL, and 100 mg/mL extracts of Yixintong tablets (the main component of which is the hawthorn leaves extract) resulted in decelerated heart rate. In addition, an intravenous injection of the 100 mg/mL extract of Yixintong tablets into the ear margin of experimental rabbits reportedly reduced their blood pressure. In a trial, the treatment group was fed with a feed prepared by adding 0.147 g/piece/day of Yixintong tablets while the control group was fed with regular feed, continuously for 7 days, and the subsequent analysis of the blood samples collected from the two groups revealed that Yixintong tablets exhibited a certain lipid-lowering effect (Qian, 2001).

### 3.2 Protection against ischemic injury

#### 3.2.1 Protective effects against myocardial ischemia

Numerous recent studies have reported that the total flavonoids isolated from hawthorn leaves exert significant protective effects against myocardial ischemia and hypoxia. For instance, the reperfusion of the total flavonoids from hawthorn leaves in distilled water at a concentration of 0.625% to myocardial ischemic rats reportedly attenuated the ST segment changes in the rat myocardium caused by the ischemia-reperfusion and elevated the nitric oxide (NO) content in the serum, which further protected the cardiomyocytes and vascular endothelial cells against the ischemia-reperfusion injury by reducing the levels of malondialdehyde (MDA) and oxygen free radical (ROS) (Kan et al., 2007). In another study, the experimental group was administered Yixintong dripping pills (the main component of which is the hawthorn leaves extract), the positive control group was administered Jiluo Yixintong tablets, and the control group was administered normal saline. The experimental group was further divided into subgroups that received Yixintong dripping pills at the doses of 25 mg/kg, 50 mg/kg, and 100 mg/kg, respectively, and each of these groups was injected with Yixintong dripping pills via duodenum and the blood samples were collected via the femoral vein. The blood sample analysis revealed that Yixintong dripping pills at the doses of 50 mg/kg and 100 mg/kg could significantly reduce the serum CK, AST, and LDH activities and preserved the ultrastructural integrity of cardiomyocytes in the animal model of acute myocardial infarction. In regard to the underlying pharmacological mechanism, it was reported that Yixintong dripping pills reduced myocardial ischemic injury by inhibiting Ca^2+^ overload (Li et al., 2003; Cui et al., 2006). Zhang et al. reported that injecting a rat model of myocardial infarction (AMI) intraperitoneally with 0.3 mg/mL, 0.6 mg/mL, and 1.2 mg/mL of the total flavonoids extract of hawthorn leaves at a dosage of 10 mL/kg body weight could effectively improve heart function, and this effect was attributed to the activation of PI3K/GSK3β/cyclin D1 signaling pathway (Zhang et al., 2021). Vitexin (VT), a flavonoid from hawthorn leaves, was demonstrated to improve the infarct size and the ventricular function of AMI in a dose-dependent manner, when used in the concentration range of 0.5–10 μM, *in vitro*. Mechanistic studies revealed that VT protects mitochondria from damage due to hypoxia by reducing the levels of ROS, in addition to reducing cardiomyocyte apoptosis by increasing the mitochondrial membrane potential and ATP content (Xue et al., 2020).

#### 3.2.2 Protective effects against cerebral ischemia

Total flavonoids from hawthorn leaves exert protective effects on ischemic brain tissue. Guangxin Miao et al. reported that 140 mg/kg/day of total flavonoids from hawthorn leaves administered through the intragastric route could reduce the inflammatory reaction and inhibited neuronal apoptosis in rats with cerebral ischemia, which protected the neural functioning of these rats with chronic cerebral ischemia (Miao et al., 2019). Moreover, in a focal cerebral ischemia-reperfusion rat model of arterial embolism, total flavonoids from hawthorn leaves reduced the expressions of MMP-9 (gelatinase B) significantly, in addition to decreasing the degradation of proteins in the vascular basement membrane and extracellular matrix, further reducing the blood-brain barrier (BBB) permeability, brain edema, and the size of cerebral infarct, thereby exerting a protective effect on the cerebrum, and this effect was attributed to the inhibition of the Caspase-3-mediated apoptotic pathway (Liu and Bao, 2006; Qu et al., 2019). Yingshi Ji et al. applied the total flavones from hawthorn leaves as a drug at high, medium, and low doses of 60 mg/kg, 30 mg/kg, and 15 mg/kg, respectively, to the rats with middle cerebral artery occlusion (MCAO), and in addition to the drug groups, a blank group and a model group that received equal volumes of normal saline were also established. The drug or saline was injected intraperitoneally (IP) once a day, and after continuous administration for 5 days, blood samples were collected under anesthesia followed by serum extraction. The subsequent analysis of the blood samples revealed that the total flavonoids from hawthorn leaves increased the activity of superoxide dismutase (SOD) while decreasing the levels of creatine kinase (CK), lactate dehydrogenase (LDH) activity, and malondialdehyde (MDA) in the serum of MCAO rats. The total flavonoid application also reduced the whole blood viscosity, plasma viscosity, and platelet aggregation in blood stasis rats. In addition, the total flavonoids from hawthorn leaves, when used in the concentration of 30–100 mg/L, reportedly inhibited the H_2_O_2_-induced apoptosis in rat pheochromocytoma cell line (PC12) in a dose-dependent manner, via the mitochondrial pathway, thereby protecting the brain cells of these model rats (Ji et al., 2005; Ji et al., 2006).

#### 3.2.3 Protective effects against ischemic liver and kidney injury

Recent studies have demonstrated that total flavonoids from hawthorn leaves exert protective effects against ischemic liver and kidney injury. For instance, the intraperitoneal injection of total flavonoids from hawthorn leaves (dosage, 60 mg/mL or 120 mg/mL) reportedly inhibited the production of free radicals by increasing the SOD activity in the liver tissue of rats with liver ischemia-reperfusion, in addition to inhibiting lipid peroxidation and reducing MDA production, thereby reducing the damage caused to the liver tissue and playing a protective role (Zhong et al., 2014). Studies on animals have revealed that injecting the rat model of renal ischemia/reperfusion (I/R) injury intravenously with total flavonoids from hawthorn leaves (dosage, 30 mg/kg or 60 mg/kg) significantly reduced the content of BUN and SCR in the serum, increase the urine volume, and reduce the production of TNF- α and IL-1, in addition to increasing the SOD activity in the renal tissue and reducing MDA production; thereby playing a protective role in renal ischemia (Chen et al., 2007).

### 3.3 Anti-anginal effects

Hawthorn leaves extract also exhibits anti-anginal effects. In a study, patients were randomly divided into two groups, and the 100 cases in the treatment group were administered Jinjia Yixintong tablets, orally, 2 tablets each time, three times a day, while the 34 cases in the control group were administered Di’ao Xinxuekang capsules, orally, 2 capsules each time, three times a day. After 4 weeks of collecting analysis data followed by statistical analysis, it was observed that the Jinjia Yixintong tablets (the main component of which is the hawthorn leaves extract) improved the cardiovascular blood supply, thereby exerting therapeutic effects on the angina (Li, 2002). Xiaoqing Wu et al. conducted a study in which they continued with the original treatment plan designed for 30 patients with coronary heart disease and angina pectoris with the addition of Xinan capsules, which were administered at a dose of 3 capsules, 3 times a day, for 4 weeks. When angina pectoris occurred, the patients were administered nitroglycerin. Finally, it was observed that Xinan capsules exerted a good effect in terms of ameliorating the ischemic ST-T changes, and this effect was correlated with the frequency and degree of the angina attacks. In addition, the amount of nitroglycerin consumed for relieving angina pectoris was observed to be reduced (Wu et al., 2002).

### 3.4 Blood glucose lowering effects

With time, novel treatment strategies for type 2 diabetes and other metabolic disorders associated with insulin resistance keep emerging (Fu et al., 2022), and increasing evidence suggests that Traditional Chinese Medicine is effective in these conditions (Zhou et al., 2021). Apigenin, quercetin, kaempferol, luteolin, and hyperoside are flavonoid compounds from hawthorn leaves that reportedly reduce blood glucose levels. Xiyun Ye used biochemical methods to detect the relevant indicators in the diabetic mouse model established via tail IV of alloxan (45 mg/kg) and reported that compared to the control group, the groups that received total flavonoids from hawthorn leaves (dosages: 0.48 g/kg, 0.96 g/kg, and 1.44 g/kg) via gavage for 30 days exhibited significantly reduced blood glucose levels in the serum of diabetic mice along with improvement in the glucose metabolism disorder caused due to hyperglycemia (Ye et al., 2005). Several recent animal experiments demonstrated that total flavonoids from hawthorn leaves significantly reduced the fasting plasma glucose levels in type 2 diabetic rats in a dose-dependent manner and also improved the pancreatic insulin-secretion function, thereby ameliorating the diabetic symptoms (Zhang and Zhang, 2015; Zhou et al., 2016; Chen et al., 2017).

### 3.5 Treatment of hyperlipidemia

Several recent studies have reported that the flavonoids present in hawthorn leaves exert lipid-lowering effects. For instance, in a study, total flavonoids from hawthorn leaves significantly reduced the levels of serum cholesterol (TC), total cholesterol (TCH), triglycerides (TG), and low-density lipoprotein cholesterol (LDL-C) in hyperlipidemia model animals. These flavonoids could also inhibit the increase in the levels of fructosamine (FA), lactate dehydrogenase (LDH), and alanine transaminase (ALT), in addition to increasing the HDL-C to TC ratio and the HDL, NO, and SOD activities. In addition, the flavonoids exhibited fat-scavenging effects on the liver tissue with fat accumulation due to diabetes. These findings indicate that the total flavonoids from hawthorn leaves significantly reduce the blood lipid levels in hyperlipidemia and exert a preventive effect on liver tissue fat accumulation (Ye et al., 2003; Ye et al., 2005; Yang et al., 2007; Liu and Yang, 2008; Zhang and Zhang, 2015). In certain experiments, simvastatin (4 mg/kg) was administered to the positive control group to determine the effect and the underlying mechanism of total flavonoids from hawthorn leaves used at the doses of 100 mg/kg, 200 mg/kg, and 400 mg/kg on hyperlipidemia in rats. The results revealed that total flavonoids from hawthorn leaves could ameliorate hyperlipidemia by inducing p-AMPK and inhibiting the p-ACC expression *in vivo*, thereby increasing fatty acid oxidation, inhibiting cholesterol synthesis, and reducing fat synthesis (Diao et al., 2020). Certain other experiments revealed the underlying mechanism of action, which was thought to be related to the improvement of abnormal blood rheology and inhibition of platelet aggregation in these animals (Yang et al., 2008). Patsouris et al. reported that total flavonoids from hawthorn leaves could activate wild-type peroxisome proliferator-activated receptors α (PPARs-α) in null mice, reducing the serum triglyceride and low-density lipoprotein levels, increasing the high-density lipoprotein levels, and improving insulin resistance, thereby realizing lipid-regulating effects (Patsouris et al., 2004). Weiwei Liang et al. administered pioglitazone hydrochloride (6 mg/kg) to the positive control group and high, medium, and low doses (12 mg/kg, 6 mg/kg, and 3 mg/kg, respectively) of total flavonoids to the treatment groups of hyperlipidemic rats, which resulted in the observation that the total flavonoids from hawthorn leaves increased the lipoprotein lipase (LPL) levels in the muscle tissue and decreased the LPL levels in the adipose tissue of these rats (Liang et al., 2010). The polysaccharide active ingredients from hawthorn leaves reportedly reduced the total cholesterol and triglycerides, thereby realizing hypolipidemic effects in hyperlipidemia model animals (Huang, 2001). Ye et al. reported that vitexin, a flavonoid from hawthorn leaves, could reduce glucose, TG, and TC levels in the serum and inhibit fat accumulation in 3T3-L1 adipocytes, thereby reducing high-fat diet (HFD)-induced obesity and adipogenesis in male C57BL/6J mice, and these effects were attributed to the activation of the AMPKα signaling pathway (Peng et al., 2019). Xinying Liu et al. applied the total flavonoids from hawthorn leaves at the concentrations of 5 μg/mL, 10 μg/mL, 50 μg/mL, 75 μg/mL, and 100 μg/mL *in vitro* and achieved fat regulation through the underlying mechanism of inhibiting the differentiation of preadipocytes into mature adipocytes and preventing the secretion of leptin and PAI-1 from mature adipocytes in a dose-dependent manner (Liu et al., 2009). Lin et al. reported that the cholesterol-lowering properties of the hawthorn leaves extract were due to its constituting triterpenoid acids, such as oleanolic acid and ursolic acid. The authors demonstrated that the extract inhibited the activity of cholesterol acyltransferase (ACAT) in Caco-2 cells, and the addition of plant sterol esters combined with triterpenoid acids further contributed to this effect (Lin et al., 2011).

### 3.6 Antioxidant effects

Hawthorn leaves extract exhibits antioxidant effects. Studies have demonstrated that compared to metformin (200 mg/kg), which was applied as the positive control, the total flavonoids from hawthorn leaves (applied at the concentrations of 50 mg/kg, 100 mg/kg, and 200 mg/kg) increased the activities of superoxide dismutase, glutathione peroxidase, and catalase, while decreasing the malondialdehyde levels in the serum of type 2 diabetes model rats in a dose-dependent manner. These results demonstrated that the total flavonoids from hawthorn leaves improved the activities of antioxidant enzymes and reduced oxidative stress damage in model rats (Zhang and Zhang, 2015; Min et al., 2017; Yang et al., 2023). In a study, the application of 40–160 mg/kg/day of total flavonoids from hawthorn leaves could activate the Nrf2-ARE pathway and thereby promote the activation and release of Nrf2, which further increased the expression of the downstream genes regulating phase II detoxification enzymes and antioxidant enzymes, thereby realizing the antioxidant stress capacity and ultimately reducing the degree of hepatic fat deposition and inflammation in the rats with steatohepatitis (Wang et al., 2018). Li et al. reported that applying 16.32–32.64 mg/kg/day of total flavonoids from hawthorn leaves significantly increased the activities of SOD and GSH PX in the heart and brain tissues and the whole blood of aging animals, reduced the MDA and LF contents, inhibited the damage due to free radicals by increasing the activities of antioxidant enzymes in the tissues, and prevented lipid peroxidation, ultimately playing the role of an anti-aging agent (Li et al., 2007). In another study, the ethanolic extract of hawthorn leaves applied at a dose range of 0.122–0.610 g/L exhibited scavenging and inhibitory effects on the generated hydroxyl radicals (OH^−^) and superoxide anions (O^2–^), and the degree of these effects was proportional to the percentage concentration of the extract used (Huang and Zeng, 1996). The polyphenolic components of hawthorn leaves are capable of scavenging the superoxide anions and serve as the main active components responsible for the antioxidant effects of hawthorn leaves. In a study, the application of 0.03–0.15 g/mL of the hawthorn leaves extract could increase the antioxidant enzyme activity, inhibit the oxidative modification of low-density lipoprotein (LDL-C), and scavenge the hydroxyl radicals, thereby exerting antioxidant effects (Yu et al., 2006). Moreover, the four different polar components of hawthorn leaves exhibit slight differences in their antioxidant capacities, with the n-butanol phase exhibiting the strongest total reducing power, the aqueous phase exhibiting a better ferrous ion-chelating ability, the ethyl acetate phase exhibiting a stronger scavenging effect on DPPH radicals and superoxide radicals, and the petroleum ether phase exhibiting the weakest antioxidant effect (Yu et al., 2006; Gong et al., 2016; Li et al., 2020).

### 3.7 Other biological activities

The ethanolic extract of hawthorn leaves (0.125–0.5 g/kg) reportedly inhibited the xylene-induced swelling of mouse ear shells, thereby exhibiting anti-inflammatory effects in a dose-dependent manner. In addition, the extract reduced the number of writhing responses in mice in response to stimulation with glacial acetic acid, thereby prolonging the ‘licking the hind foot’ duration and the tail flicking duration among the mice in the hot plate model and hot water tail withdrawal model. The extract also exhibited an analgesic effect in a dose-dependent manner (Quan and Guan, 2006; Wang et al., 2012). The bioactivities of the different chemical components of hawthorn leaves are listed in Table 1.

**TABLE 1:** Bioactivity of different chemical composition.

Bioactivity	Chemical composition	Mechanism	Ref
Lower blood pressure	Total Flavonoids, Proanthocyanidins, Triterpenoid Acid Extracts, and Polysaccharides	Induce vascular smooth muscle cell and endothelial cell dependent relaxation leading to peripheral vasodilation	Huang (2001); Chang et al. (2002)
	Yixintong tablets (The main components are total flavonoids)	Counteracts the actions of acetylcholine and epinephrine to adjust cardiac contractility and heart rate	Qian (2001)
Protecting myocardial ischemia	Total Flavonoids	Reduce ROS production, increase NO levels, and inhibit MDA production, which in turn protect cardiomyocytes and vascular endothelial cells	Kan et al. (2007)
	Yixintong capsules (The main components are total flavonoids)	Inhibition of Ca^2+^ overload thereby reduces myocardial ischemic injury	Li et al. (2003), Cui et al. (2006)
	Vitexin (Flavonoid)	Reduce ROS production, Improve mitochondrial activity, mitochondrial membrane potential and ATP content, Significantly increase Mfn2 expression and reduce Drp1 recruitment to mitochondria, alleviate mitochondrial damage and then reduce cardiomyocyte apoptosis	Xue et al. (2020)
Protecting cerebral ischemia	Total Flavonoids	Reduce ROS production, attenuate inflammatory response and inhibit neuronal apoptosis, protecting brain cells	Miao et al. (2019)
Protecting ischemic liver and kidney injury	Total Flavonoids	Enhance SOD activity and inhibit ROS production	Zhong et al. (2014)
Protecting angina	Jinjia Yixintong tablets (The main components are total flavonoids)	Improve blood supply to the cardiovascular, Relief of spasm of coronary arteries, invigorating blood circulation and eliminating stasis, Tongmai depression	Li et al., 1988; Li (2002)
	Xinan capsules (The main components are total flavonoids)	Lower blood pressure, lipids and blood glucose, improve the frequency and extent of angina attacks	Wu et al. (2002)
Hypoglycemic	Total Flavonoids	Increase pancreatic insulin secretion	Zhang and Zhang (2015), Zhou et al. (2016), Chen et al. (2017)
Treatment of hyperlipidemia	Total Flavonoids	Decrease TC, TCH, TG and LDL-C, inhibit FA, LDH, and ALT and increase the HDL-C to TC ratio, elevate HDL, NO and SOD activity levels, Clean fat accumulation in liver tissue caused by diabetes, Induced p-AMPK, Inhibit the expression of p-ACC, Modulation of LPL; Inhibit secretion of Leptin, PAI-1	Ye et al. (2003), Ye et al. (2005), Yang et al. (2007), Liu and Yang (2008), Zhang and Zhang (2015), Diao et al. (2020)
	Vitexin (Flavonoid)	Lowering glucose, TG, TC and inhibiting fat accumulation	Peng et al. (2019)
	Triterpenoid acids (Oleanolic acid and Ursolic acid)	Inhibit the activity of ACAT	Lin et al. (2011)
Antioxidant	Total Flavonoids	Activation of the Nrf2-ARE pathway, increase SOD and GSH-Px viability and decrease MDA and LF content	Li et al. (2007), Zhang and Zhang (2015), Min et al. (2017), Wang et al. (2018)
Anti-Inflammatory	Ethanol extract (90.8% flavonoids)	Related to Ca^2+^ antagonism	Wang et al. (2012)

## 4 Quality standard

The quality standard of hawthorn leaves was documented first in the 2005 edition of Chinese Pharmacopoeia, and it has since remained the same until the 2020 edition of Chinese Pharmacopoeia was published. According to the 2020 edition of Chinese Pharmacopoeia, the standard for the differentiation of hawthorn leaves is rutin with hyperoside, with the moisture examined not exceeding 12.0% and the acid-insoluble ash not exceeding 3.0%. In addition, the leachates shall be determined through the light cold leaching method, using dilute ethanol as the solvent, and the determined levels not exceeding 20.0%. The specified anhydrous rutin content in hawthorn leaves shall not be less than 7.0%, while the hyperoside content shall not be less than 0.050% when calculated on a dry basis.

### 4.1 Chemical composition and content determination

In the Chinese Pharmacopoeia 2020, rutin and hyperoside are listed as the content determination indicators for hawthorn leaves, and both of these are flavonoids. The content of hawthorn leaf components studied in other literature is shown in Table 2.

**TABLE 2:** Chemical composition content.

No.	Name	Category	Method	Content (%)	Ref
1	vitexin glucoside	Flavonoid	UPLC	2.5000	Xu (2019)
2	vitexin rhamnoside	Flavonoid	UPLC	12.0000	Xu (2019)
3	vitexin	Flavonoid	UPLC	0.2000	Xu (2019)
			HPLC	0.0960–0.7890	Chen, 2006; Ying (2007), Yuan and Li (2007)
4	hyperoside	Flavonoid	UPLC	0.0700	Xu (2019)
			HPLC	0.3490	Ying (2007)
5	2″-O-rhamnovitexin	Flavonoid	HPLC	1.9245	Chen (2006)
6	rutin	Flavonoid	HPLC	0.0510–0.3410	Ying, 2007; Wang et al. (2013)
7	quercetin	Flavonoid	HPLC	0.3690–0.0061	Ying, 2007; Yuan and Li (2007)
8	isorhamnetin	Flavonoid	HPLC	0.2020	Yuan and Li (2007)
9	vitexin-4′-o-glucoside	Flavonoid	HPLC	4.8500	Ying (2007)
10	vitexin-2″-o-rhamnoside	Flavonoid	HPLC	6.1700	Ying (2007)
11	isoquercetin	Flavonoid	HPLC	0.1980	Ying (2007)
12	epicatechin	Flavonoid	HPLC	0.0239	Liu and Yu (2007)
13	ursolic acid	Terpenoids	HPLC	3.4860–3.5210	Lu and Wei (2008)
14	chlorogenic acid	Organic acids	HPLC	0.0316–0.0327	Liu and Yu, 2007; Ying (2007)
15	ferulic acid	Organic acids	HPLC	0.1325	Liu and Yu (2007)
16	2, 7 (14), 10-bisabolatrien-1-ol-4-one	Volatile oil	GC-MS	18.4200	Cui et al. (2014)
17	nezukol	Volatile oil	GC-MS	6.2500	Cui et al. (2014)
18	transsabinene hydrate	Volatile oil	GC-MS	4.1100	Cui et al. (2014)
19	citronellyl propanoate	Volatile oil	GC-MS	2.8000	Cui et al. (2014)
20	zonarene	Volatile oil	GC-MS	2.6500	Cui et al. (2014)
21	(5E, 9E)-farnesyl acetone	Volatile oil	GC-MS	2.5000	Cui et al. (2014)

#### 4.1.1 Flavonoids

Among the 12 flavonoids in hawthorn leaves, Vitexin glucoside, Vitexin rhamnoside, Vitexin, and hyperin are present at a content percentage of at least 2.5000%, 12.0000%, 0.2000%, and 0.0700%, respectively, as determined using UPLC. The vitexin content in the hawthorn leaves extract, as determined using HPLC, was in the range of 0.0960%–0.7890%, and the content range for rutin and quercetin was 0.0510%–0.3410% and 0.3690%–0.0061%, respectively. In addition, the contents of hyperin, 2″-O-rhamnovitexin, isorhamnetin, vitexin-4′-O-glucoside, vitexin-2″-O-rhamnoside, isoquercetin, and epicatechin were not less than 0.3490%, 1.9245%, 0.2020%,4.8500%, 6.1700%, 0.1980%, and 0.0239%, respectively.

#### 4.1.2 Terpenoids

The contents of terpenoids in hawthorn leaves are relatively less reported. The content of ursolic acid in the hawthorn leaves extract, determined using HPLC, was in the range of 3.4860%–3.5210%.

#### 4.1.3 Organic acids

The contents of organic acids in hawthorn leaves were analyzed using HPLC and determined to be in the range of 0.0316%–0.0327% for chlorogenic acid and at least 0.1325% for ferulic acid.

#### 4.1.4 Volatile oils

The contents of the major volatile oil components in hawthorn leaves were determined using gas chromatography-mass spectrometry, which revealed 18.4200% of 2,7(14),10-bisabolatrien-1-ol-4-one, 6.25% of nezukol, 4.11% of transsabinene hydrate, 2.8% of citronellyl propanoate, 2.65% of zonarene, and 2.5% of (5E,9E)-farnesyl acetone.

Hawthorn leaves contains several components, and whether a single quality control index would ensure the quality of medicinal materials remains debatable to date. Therefore, it is necessary to conduct a further detailed study on the other components of hawthorn leaves.

### 4.2 Fingerprinting studies

Recently, several research groups conducted fingerprinting studies on hawthorn leaves. For instance, Jing Gao et al. used HPLC to establish the fingerprint for hawthorn leaves and also compared and analyzed four different extraction fractions of hawthorn leaves. The authors reported that the number and variety of the common peaks obtained for the different extraction fractions were slightly different. The authors also compared the fingerprints of the hawthorn leaves samples from various production regions and identified quite different fingerprint profiles. The total content of the main components in the hawthorn leaves samples from the main production region remained relatively stable and at a high level overall. However, the quality of hawthorn leaves was observed to be significantly different when the samples were obtained from different producing regions in Guangdong and Guangxi (Gao et al., 2018a; Gao et al., 2018b).

The characteristic fingerprint peaks of hawthorn leaves samples from different producing regions have been detected using HPLC, UPLC, and RP-HPLC, although it remains debatable whether the hawthorn leaves samples from different producing regions present significant differences in their fingerprint peaks (Liu and Yu, 2007; Hu et al., 2017; Guan et al., 2020). According to the composition studies, phenolic acids are the major differential markers between wild and commercially available hawthorn leaves, and the contents of chlorogenic acid, rutin, and hyperoside differ greatly among the hawthorn leaves varieties produced at Chengde, Shandong, and Liaoning (Wang et al., 2011; Hu et al., 2017). Studies on the hawthorn leaves medicinal botanical drug samples collected from 11 producing regions, including Hefei, Jincheng, Shanxi, and Beijing in China, revealed huge differences in the composition of hawthorn leaves among different producing regions, with greater chromatographic peaks detected in the hawthorn leaves samples from Jincheng and Shanxi in China (Li et al., 2016). The fingerprints of the hawthorn leaves samples from northern and southern China also differed in terms of the number of peaks, although the differences in the fingerprints were not evident prior to 13.05 min. Moreover, under the same chromatographic conditions, the southern hawthorn leaves exhibited greater peaks after 17.39 min (Xu et al., 2017).

Yilong Du used HPLC to determine the contents of six index components, namely, vitexin rhamnoside, vitexin glucoside, chlorogenic acid, rutin, vitexin, and hyperoside, in hawthorn leaves at different harvesting times. The authors reported that the peak area obtained for each component varied with the month of harvest. Wei Zheng et al. obtained the chemical information of hawthorn leaves using ultra-high-performance liquid chromatography and quadrupole time-of-flight mass spectrometry (UHPLC-Q-TOF-MS), following which they performed a principal component analysis (PCA) to compare the chemical components in the hawthorn leaves harvested in different months. Finally, the authors reported that the harvest season of hawthorn leaves exerted a significant impact on its chemical composition, with relatively high contents of the different components observed in the autumn harvest (Zheng et al., 2022). UV–Vis spectrophotometric studies also revealed evident differences in the total flavonoid contents in the Chengde hawthorn leaves samples harvested at different times. The determination of the contents of the six index components and the content of total flavonoids, including vitexin rhamnoside, in hawthorn leaves revealed that the contents and the peak areas obtained for each component were the highest in May, followed by August, and the lowest contents and peak areas were noted for November (Du, 2013). These findings suggest that those working in the field of Traditional Chinese Medicine should ensure the quality of hawthorn leaves by using the distinguishing of the different components of authentic and counterfeit hawthorn leaves as the detection index and harvesting the samples in autumn to maximize the contents of the components in hawthorn leaves. In addition, since the quality of hawthorn leaves from different habitats is different, further detailed research must be conducted in this regard as well.

### 4.3 Pesticide residues

Yuanxi Liu et al. detected up to 19 pesticides in six batches of hawthorn leaves using gas chromatography tandem mass spectrometry and liquid chromatography tandem mass spectrometry, and on average, 13 pesticides were detected in each batch. All of these pesticides detected are common pesticides used in China and were detected without restriction (Liu et al., 2022). Among these, five pesticides, namely, carbendazim, imidacloprid, pyrazolyl axetil, hexazol, and phentermine methiconazole, were detected in 100% of the six batches of hawthorn leaves, while 15 pesticides were detected in over or equal to 50% of the six batches, with three batches of sample nitrile, one batch of sample tebuconazole, one batch of sample chlorpyrifos, and one batch of sample phentermine methiconazole being over-represented. Pesticides cause serious harm to the human body and, therefore, pesticide residues in hawthorn leaves should be strictly controlled to ensure the safety of hawthorn leaves users.

## 5 Conclusion

The present report provides a comprehensive review of the chemical composition, bioactivities, and quality standard of hawthorn leaves, a commonly used Chinese herbal medicine recognized and used for lowering the blood levels of lipids and antithrombosis, anti-hypotension, anti-ischemia, anti-arrhythmia, and anti-inflammatory effects. So far, over a hundred compounds, including flavonoids, terpenes, lignans, organic acids, and nitrogenous compounds, have been isolated from hawthorn leaves. Among the main components of hawthorn leaves are flavonoids, which exhibit a wide range of pharmacological effects and also play important roles in tissues by elevating the activity of antioxidant enzymes in the tissues, inhibiting the damage caused due to free radicals, preventing lipid peroxidation, and inhibiting cell apoptosis. Hawthorn leaves are abundant in resources, inexpensive, and readily available in China, although a considerable variation has been noted in the composition of hawthorn leaves samples from different producing regions. The flavonoids such as vitexin-4′-O-glucoside, vitexin-2″-o-rhamnoside, vitexin glucoside, and vitexin rhamnoside account for a higher proportion in hawthorn leaves and exhibit evident pharmacological effects, and these could be used as indices for controlling the quality standards of hawthorn leaves. Overall, the present report provides a systematic review that would serve as a scientific basis for the detailed research on the development of hawthorn leaves based on recent advances in the knowledge of its chemical composition, biological activities, and quality standards.
