# Supplementary material for: Chemical composition, biological activities, and quality standards of hawthorn leaves used in traditional Chinese medicine: a comprehensive review
Source: Front Pharmacol. 2023 Oct 20;14:1275244. doi: 10.3389/fphar.2023.1275244 (PMC10623334; doi:10.3389/fphar.2023.1275244)
Supplement: Supplementary file 1 [file Table1.docx]

**Supplementary Table**

**Table S1 Quercetin flavonoids**

| **No.** | **Name** | **Ref.** |
| --- | --- | --- |
| 1 | spiraeside | (Sun et al., 2015) |
| 2 | quercetin | (Ding et al., 1990) |
| 3 | hyperoside | (Ding et al., 1990) |
| 4 | glogoside | (Chen, 2006) |
| 5 | 3-*O*-β-D-glucopyanosyl quercetin | (Chen et al., 2006) |
| 6 | bioquercetin | (Wang, 2020) |
| 7 | isoquercitrin | (Du et al., 2022) |
| 8 | rutin | (Si et al., 1998) |
| 9 | (+)-taxifolin | (Shahat et al., 1998) |
| 10 | (+)-taxifolin-3-O-arabinopyranoside-3-O-arabinopyranoside | (Shahat et al., 1998) |
| 11 | (+)-taxifolin-3-O-xylopyranoside | (Shahat et al., 1998) |
| 12 | 3,4',5,8-tetrahyduoxyl-flavone-7-O-glucosyl | (Chen, 2006) |
| 13 | 3-O-β-D-glucopyanosyl(6→1)-α-L-rhamnosyl quercetin | (Zhang and Xu, 2001a) |
| 14 | 3-O-β-D-galacopyanosyl(6→1)-α-L-rhamnosyl quercetin] | (Zhang and Xu, 2001a) |
| 15 | 3'-O-arabinosyl quercetin | (Chen, 2006) |

**Table S2 Kaempferol flavonoids**

| **No.** | **Name** | **Ref.** |
| --- | --- | --- |
| 16 | kaempeforl | (Zhang and Xu, 2001a) |
| 17 | 8-methoxy kaempeforl | (Zhang and Xu, 1999a) |
| 18 | 8-methoxy keampeforl-3-O-glueoside | (Zhang and Xu, 1999a) |
| 19 | herbacetin | (Wang, 2020) |
| 20 | 8-methoxy keampeforl neohesperidoside | (Zhao et al., 2002) |
| 21 | keampeforl-3-O-neohesperidoside | (Zhao et al., 2002) |
| 22 | pinnatifidin | (Chen, 2006) |
| 23 | 7-O-α-L-rhamnoside-3-O-β-D-glueopynaosyl keampefrol | (Zhang and Xu, 2001a) |

**Table S3 Apigenin flavonoids**

| **No.** | **Name** | **Ref.** |
| --- | --- | --- |
| 24 | 5,4'-dihydorxl-7-O-rhamnosyl apigenin | (Zhang and Xu, 1999b) |
| 25 | vitexin-4',7-diglucoside | (Zhang and Xu, 1999b) |
| 26 | vitexin | (Ding et al., 1990) |
| 27 | 2''-O-rhamnosyl vitexin | (Ding et al., 1990) |
| 28 | vitexin-2''-O-rhamnosyl | (Xu et al., 1985) |
| 29 | vitexin-4'-O-rhamnoside | (Liu et al., 2007) |
| 30 | vitexin-2''-O-(4'''-O-acetyl) rhamnoside | (Liu et al., 2007) |
| 31 | vitexin-2''-O-glucosyl | (Hao, 2008) |
| 32 | iso-vitexin | (Xu et al., 1985) |
| 33 | isovitexin-2''-O-rhamnoside | (Zhang and Xu, 1999a) |
| 34 | cratenacin | (Zhang and Xu, 1999b) |
| 35 | isoschaftoside | (Nikolov and Litvinenko, 1973) |
| 36 | pinnatifinoside(B) | (Zhang and Xu, 2001b) |
| 37 | vicenin 3(6-C-glueosyl-8-C-xylosyl Apigenin) | (Chen, 2006) |
| 38 | icariin | (Zhang and Xu, 1999a) |
| 39 | neoisoschaftoside | (Nikolov and Litvinenko, 1973) |
| 40 | pinnatifinoside(C) | (Zhang and Xu, 2001b) |
| 41 | pinnatifinoside(D) | (Zhang and Xu, 2001b) |
| 42 | pinnatifinoside(I) | (Zhang and Xu, 2001b) |
| 43 | schaftosise | (Nikolov and Litvinenko, 1973) |
| 44 | 4''-O-glucosyl vitexin | (Hao, 2008) |
| 45 | neoschaftoside | (Nikolov and Litvinenko, 1973) |
| 46 | 7-O-glueoside apigenin | (Chen, 2006) |
| 47 | pinnatifinoside(A) | (Zhang and Xu, 2001b) |
| 48 | vitexin-6''-O-acetyl | (Xu et al., 1985) |
| 49 | 8-C-(6''-O-acetyl-4''-O-rhamnosyl)-glucosyl apigenin | (Chen, 2006) |
| 50 | vicenin1 (6-C-xylsoyl-8-C-glucosyl apigenin) | (Chen, 2006) |
| 51 | 3''-O-acetyl vitexin | (Chen, 2006) |
| 52 | 8-C-β-D-(2''-O-acetyl)-glucofunoside apigenin | (Chen, 2006) |
| 53 | 6-C-hexosyl-8-C-pentosyl apigenin | (Chen, 2006) |
| 54 | vicenin 2(6,8-diglucosyl apigenin) | (Chen, 2006) |
| 55 | 8-C-glucosyl-rhamnosl apigenin | (Chen, 2006) |

**Table S4 Luteolin flavonoids**

| **No.** | **Name** | **Ref.** |
| --- | --- | --- |
| 56 | luteolin-3',7-diglucoside | (Zhang and Xu, 1999a) |
| 57 | orientin | (Nikolov and Litvinenko, 1973) |
| 58 | luteolin-7-O-glucoside | (Zhang and Xu, 1999b) |
| 59 | isoorientin | (Nikolov and Litvinenko, 1973) |
| 60 | 2''-O-rhamnoside orientin | (Chen, 2006) |
| 61 | 2''-O-rhamnoside-isoorientin | (Chen, 2006) |

**Table S5 Dihydroflavonoid flavonoids**

| **No.** | **Name** | **Ref.** |
| --- | --- | --- |
| 62 | naringenin-5,7-O-diglucoside | (Zhang and Xu, 1999b) |
| 63 | eriodictyol-5,3'-diglucoside | (Zhang and Xu, 1999b) |
| 64 | eriodictyol-7,3'-diglucoside | (Zhang and Xu, 1999a) |

**Table S6 Flavans and their polymers**

| **No.** | **Name** | **Ref.** |
| --- | --- | --- |
| 65 | cyanidin | (Zhang and Xu, 1999a) |
| 66 | (-)-epicatechin | (Zhang and Xu, 1999a) |
| 67 | (+)-catechin | (Zhang and Xu, 1999a) |
| 68 | achromatopsia | (Zhang and Xu, 1999a) |
| 69 | pelargonidin | (Zhang and Xu, 1999a) |
| 70 | dimeric colorless cyanidin | (Zhang and Xu, 1999a) |
| 71 | cianidanol | (Chen, 2006) |
| 72 | cinchonain Ib | (Wang, 2020) |
| 73 | epicatechin-(4β→8)-epicatechin-(4β→6)-epicatechin | (Svedstrom et al., 2002) |
| 74 | epicatechin-(4β→6)-epicatechin-(4β→8)-epicatechin | (Svedstrom et al., 2002) |

**Table S7 Anthocyanins**

| **No.** | **Name** | **Ref.** |
| --- | --- | --- |
| 75 | proanthocyanidin A2 | (Svedstrom et al., 2002) |
| 76 | procyanidin B2 | (Svedstrom et al., 2002) |
| 77 | procyanidin B4 | (Svedstrom et al., 2002) |
| 78 | procyanidin B5 | (Svedstrom et al., 2002) |
| 79 | procyanidin C1 | (Svedstrom et al., 2002) |
| 80 | procyanidin E1 | (Svedstrom et al., 2002) |
| 81 | procyanidin D1 | (Svedstrom et al., 2002) |

**Table S8 Triterpenoids and Steroids**

| **No.** | **Name** | **Ref.** |
| --- | --- | --- |
| 82 | 2α,3β,19α-trihydroxyl ursolic acid | (Song et al., 2006) |
| 83 | cycloartenol | (Garcia et al., 1997) |
| 84 | butyrospermol | (Garcia et al., 1997) |
| 85 | 24-methylene-24-dihydrolanosterol | (Garcia et al., 1997) |
| 86 | 18,19-seco,2α,3β-dihydroxy-19-oxo-urs-11,13(18)-dien-28-oic acid | (Huang et al., 2010) |
| 87 | oleanlic acid | (Zhang and Xu, 1999a) |
| 88 | lupeol | (Gao, 2009) |
| 89 | euscaphic acid | (Wang, 2020) |
| 90 | tormentic acid | (Duan et al., 2021) |
| 91 | ursolic acid | (Si et al., 1998) |
| 92 | pomolic acid | (Wang, 2020) |
| 93 | corosolic acid | (Chen et al., 2008) |
| 94 | crataegolic acid | (Duan et al., 2021) |
| 95 | β-sitosterol | (Hao, 2008) |
| 96 | α-amyrin | (Gao, 2009) |
| 97 | daucosterol | (Si et al., 1998) |
| 98 | (S)-linalyl rutinoside | (Duan et al., 2021) |
| 99 | (Z)-3-hexenyl -O-β-D-glucoside | (Wang, 2020) |
| 100 | (3S, 6S)-cis-linalool-3,7-oxide-β-D-glucopyranoside | (Wang, 2020) |
| 101 | byzantionoside B | (Wang, 2020) |
| 102 | (6R,7E,9R)-9-hydroxy-4,7-megastigmadien-3-one 9-O-β-D-glucopyranoside | (Duan et al., 2021) |
| 103 | urs-12-ene-3β,28-diol | (Shi and Ding, 2000) |
| 104 | 2α,3β,12β,19α- tetrahydroxyuracil-13β,28- lactone | (Duan et al., 2021) |

**Table S9 Monoterpenes and Sesquiterpenes**

| **No.** | **Name** | **Ref.** |
| --- | --- | --- |
| 105 | 3,9-dihydroxymegastigma-5-ene | (Huang et al., 2010) |
| 106 | (3S,5R,6R,7E)-megatsigmane-7-ene-3-hydroxy-5,6-epoxy-9-O-β-D-glucopyranoside | (Huang et al., 2010) |
| 107 | (3R,5S,6S,7E,9S)-megatsigmane-7-ene-3,5,6,9-tetrol-9-O-β-D-glucopyranoside | (Song et al., 2011) |
| 108 | linarionoside C | (Gao et al., 2010) |
| 109 | linarionoside A | (Gao et al., 2010) |
| 110 | linarionoside B | (Gao et al., 2010) |
| 111 | (6S,7E,9R)-6,9-dihydroxy-4,7-megastigmadien-3-one -9-O-[β-D-xylopyranosyl-(1''→6')-β-D-glucopyranoside] | (Song et al., 2011) |
| 112 | icariside B_6_ | (Gao et al., 2010) |
| 113 | pisumionoside | (Gao et al., 2010) |
| 114 | (3S,5R,6R,7E,9R)-3,6-epoxy-7-megatsigmane-5,9-diol-9-O-β-D-glucopyranoside | (Gao et al., 2010) |
| 115 | (6S,7E,9R)-roseoside | (Gao et al., 2010) |
| 116 | (6R,9R)-3-oxo-α-ionol-9-O-β-D-glucopyranoside | (Gao et al., 2010) |
| 117 | (5Z)-6-[5-(2-hydroxypropan-2-yl)-2-methyltetrahydrofur-an-2-yl]-3-methylhexa-1,5-dien-3-ol | (Song et al., 2011) |
| 118 | (5Z)-6-[5-(2-O-β-D-glucopyranosyl-propan-2-yl)-2-methyltetrahydrofur-an-2-yl]-3-methylhexa-1,5-dien-3-ol | (Song et al., 2011) |
| 119 | 5-Ethenyl-2-[2-O-β-D-glucopyranosyl-(1''→6')-β-D-glucopyranosyl-propan-2-yl]-5-methyltetrahydrofuran-2-ol | (Song et al., 2011) |
| 120 | 4-[4β-O-β-D-xylopyranosyl-(1''→6')- β-D-glucopyranosyl-2,6,6-trimethyl-1-cyclohexen-1-yl]-butan-2-one | (Song et al., 2011) |
| 121 | (3S,9R)-3,9-dihydroxy-megastigman-5-ene-3-O-primeveroside | (Song et al., 2011) |
| 122 | (3R,5S,6S,7E,9S)-megastiman-7-ene-3,5,6,9-tetrol | (Song et al., 2011) |
| 123 | 3β-D-glucopyranosyloxy-β-ionone | (Gao et al., 2010) |

**Table S10 Lignan Compounds**

| **No.** | **Name** | **Ref.** |
| --- | --- | --- |
| 124 | 2,3-dihydro-2-(4’-O-β-D-glucopyranosyl-3’-methoxy-phenyl)-3-hydroxymethyl-5-(3-hydroxpropyl)-7-methoxybenzofuran | (Hao, 2008) |
| 125 | Shanyenoside A | (Chen et al., 2006) |
| 126 | (7S,8R)-urolignoside | (Gao et al., 2010) |
| 127 | (-)-2a-O-(β-D-Glucopyranosyl)-lyoniresinol | (Gao et al., 2010) |
| 128 | tortoside A | (Gao et al., 2010) |
| 129 | yerbascoside | (Gao et al., 2010) |
| 130 | acernikol-4''-O-β-D-glucopyranoside | (Gao et al., 2010) |
| 131 | erythron-1-(4-O-β-D-Glucopyranosyl-3-methoxyphenyl)-2-[4-(3-hydroxypropyl)-2,6-dimethoxyphenoxy]-1,3-propanediol | (Gao et al., 2010) |
| 132 | (7S,8R)-5-methoxydihydrodehydrodiconiferyl alcohol-4-O-β-D-glucopyranoside | (Gao et al., 2010) |

**Table S11 Organic Acid Compounds**

| **No.** | **Name** | **Ref.** |
| --- | --- | --- |
| 133 | chlorogenic acid | (Gao, 2009) |
| 134 | caffeic acid | (Wang et al., 2011) |
| 135 | benzoic acid | (Huang et al., 2010) |

**Table S12 Amino Acid Content**

| **No.** | **Name** | **Ref.** |
| --- | --- | --- |
| 136 | glutamate | (Liang et al., 1996) |
| 137 | leucine | (Liang et al., 1996) |
| 138 | aspartic acid | (Liang et al., 1996) |
| 139 | alanine | (Liang et al., 1996) |
| 140 | glycine | (Liang et al., 1996) |
| 141 | valine | (Liang et al., 1996) |
| 142 | lysine | (Liang et al., 1996) |
| 143 | arginine | (Liang et al., 1996) |
| 144 | phenylalanine | (Liang et al., 1996) |
| 145 | threonine | (Liang et al., 1996) |
| 146 | serine | (Liang et al., 1996) |
| 147 | isoleucine | (Liang et al., 1996) |
| 148 | proline | (Liang et al., 1996) |
| 149 | tyrosine | (Liang et al., 1996) |
| 150 | histidine | (Liang et al., 1996) |
| 151 | methionine | (Liang et al., 1996) |
| 152 | cystine | (Liang et al., 1996) |

**Table S13 Nitrogen containing compounds**

| **No.** | **Name** | **Ref.** |
| --- | --- | --- |
| 153 | ethylamine | (FOSTER and HOBBS, 1994) |
| 154 | isobutylamine | (FOSTER and HOBBS, 1994) |
| 155 | dimethylamine | (FOSTER and HOBBS, 1994) |
| 156 | trimethylamine) | (FOSTER and HOBBS, 1994) |
| 157 | isoamylamine | (FOSTER and HOBBS, 1994) |
| 158 | ethanolamine | (FOSTER and HOBBS, 1994) |
| 159 | choline | (FOSTER and HOBBS, 1994) |
| 160 | acetylcholine | (FOSTER and HOBBS, 1994) |
| 161 | O-methoxyphenethylamine | (FOSTER and HOBBS, 1994) |
| 162 | tyramine | (FOSTER and HOBBS, 1994) |
| 163 | phenylethylamine | (FOSTER and HOBBS, 1994) |
| 164 | spermindine | (FOSTER and HOBBS, 1994) |

**Table S14 Trace Elements**

| **No.** | **Name** | **Content (mg/kg)** | **Ref.** |
| --- | --- | --- | --- |
| 165 | Cu | 6.3 | (Zhao et al., 1992) |
| 166 | Zn | 11.4 | (Zhao et al., 1992) |
| 167 | Fe | 429 | (Zhao et al., 1992) |
| 168 | Mn | 45.5 | (Zhao et al., 1992) |
| 169 | Sr | 92.4 | (Zhao et al., 1992) |
| 170 | Ca | 40200 | (Zhao et al., 1992) |
| 171 | Mg | 4600 | (Zhao et al., 1992) |
| 172 | K | 11400 | (Zhao et al., 1992) |
| 173 | Na | 67 | (Zhao et al., 1992) |
| 174 | V | 1.3 | (Zhao et al., 1992) |
| 175 | P | 2150 | (Zhao et al., 1992) |
| 176 | Co | 0.63 | (Zhao et al., 1992) |
| 177 | Ni | ＜2 | (Zhao et al., 1992) |
| 178 | Cr | ＜2 | (Zhao et al., 1992) |
| 179 | Mo | ＜2 | (Zhao et al., 1992) |
| 180 | Al | 344 | (Zhao et al., 1992) |
| 181 | Ti | 10.3 | (Zhao et al., 1992) |
| 182 | Ba | 141 | (Zhao et al., 1992) |
| 183 | Li | ＜0.3 | (Zhao et al., 1992) |
| 184 | Be | ＜1 | (Zhao et al., 1992) |
| 185 | Bi | ＜10 | (Zhao et al., 1992) |
| 186 | Pb | ＜6 | (Zhao et al., 1992) |
| 187 | Cd | ＜0.5 | (Zhao et al., 1992) |
| 188 | As | 0.37 | (Zhao et al., 1992) |
| 189 | Hg | 0.08 | (Zhao et al., 1992) |

**Table S15 Other classes of compounds**

| **No.** | **Name** | **Ref.** |
| --- | --- | --- |
| 190 | (Z)-3-hexenyl-O-β-D-glucopyranosyl-(1''→6')-β-D-glucopyranoside | (Song et al., 2011) |
| 191 | (Z)-3-hexenyl-O-β-D-xylopyranosyl-(1''→6')-β-D-glucopyranoside | (Song et al., 2011) |
| 192 | (Z)-3-hexenyl-O-β-D-rhamnopyranosyl-(1''→6')-β-D-glucopyranoside | (Song et al., 2011) |
